# Demographic History of European Populations of *Arabidopsis thaliana*

**DOI:** 10.1371/journal.pgen.1000075

**Published:** 2008-05-16

**Authors:** Olivier François, Michael G. B. Blum, Mattias Jakobsson, Noah A. Rosenberg

**Affiliations:** 1 Institut National Polytechnique de Grenoble, Grenoble, France; 2 Centre National de la Recherche Scientifique, TIMC-IMAG, Faculty of Medicine, La Tronche, France; 3 Department of Human Genetics, Center for Computational Medicine and Biology, University of Michigan, Ann Arbor, Michigan, United States of America; 4 The Life Sciences Institute, University of Michigan, Ann Arbor, Michigan, United States of America; University of Aarhus, Denmark

## Abstract

The model plant species *Arabidopsis thaliana* is successful at colonizing land that has recently undergone human-mediated disturbance. To investigate the prehistoric spread of *A. thaliana*, we applied approximate Bayesian computation and explicit spatial modeling to 76 European accessions sequenced at 876 nuclear loci. We find evidence that a major migration wave occurred from east to west, affecting most of the sampled individuals. The longitudinal gradient appears to result from the plant having spread in Europe from the east ∼10,000 years ago, with a rate of westward spread of ∼0.9 km/year. This wave-of-advance model is consistent with a natural colonization from an eastern glacial refugium that overwhelmed ancient western lineages. However, the speed and time frame of the model also suggest that the migration of *A. thaliana* into Europe may have accompanied the spread of agriculture during the Neolithic transition.

## Author Summary

The demographic forces that have shaped the pattern of genetic variability in the plant species *Arabidopsis thaliana* provide an important backdrop for the use of this model organism in understanding the genetic determinants of plant natural variation. We investigated the demographic history of *A. thaliana* using novel population-genetic tools applied to a combination of molecular and geographic data. We infer that *A. thaliana* entered Europe from the east and spread westward at a rate of ∼0.9 kilometers per year, and that its population size began increasing around 10,000 years ago. The “wave-of-advance” model suggested by these results is potentially consistent with the pattern expected if the species colonized Europe as the ice retreated at the end of the most recent glaciation. Alternatively, it is also compatible with the possibility that *A. thaliana*—a weedy species—may have spread into Europe with the diffusion of agriculture, providing an example of the phenomenon of “ecological imperialism” described by A. Crosby. In this framework, just as weeds from Europe invaded temperate regions worldwide during European human colonization, weeds originating from the source region of farming invaded Europe as a result of the disturbance caused by the spread of agriculture.

## Introduction

*Arabidopsis thaliana* is an important model organism for plant biology, serving as a focal species for studies of plant physiology, molecular biology, and genetics [1]–[4]. Its use as a model species is facilitated by its short generation time in the laboratory, its production of large numbers of seeds, and its reproduction primarily by self-fertilization.

Many of the same traits that contribute to the utility of *A. thaliana* as a model organism are important in determining the niche of the species in its natural environment. Its rapid flowering, self-fertilization, and extensive seed production are characteristic of colonizing species that grow in open or recently disturbed habitats [5],[6]. From an ecological standpoint, due to its status as a colonizing species, *A. thaliana* can be viewed as a weed.

*A. thaliana* is frequently described as native to the Eurasian landmass [6],[7], and in recent times it has been among the group of weeds from Europe that have invaded North America and Australia since the time of European colonization [8],[9]. However, relatively little is known about the prehistoric spread of the species into Europe. Because pollen from *A. thaliana* is very similar to that of many other species from the Brassicaceae family [10], it is often undetectable in surveys of past plant geographic distributions. Thus, investigations of patterns of present-day genetic variation have provided an important alternative method for understanding the recent history of the species.

Most European species are believed to have been restricted to southern refugia at the height of glaciation ∼18,000 BP—many in the peninsulas of Iberia, Italy, and the Balkans, and some near the Caucasus region and the Caspian Sea [11]–[13]. When the climate warmed and the ice retreated, these species expanded their ranges northwards, starting ∼16,000 BP [14]. For *Arabidopsis thaliana*, on the basis of population-genetic data, Sharbel et al. [15] proposed a scenario of post-glacial re-colonization of Europe from two refugia, one in the Iberian Peninsula and the other in central Asia, followed by admixture of the two ancestral populations in central and eastern Europe. However, contradicting the predictions of this model, Schmid et al. [16] found that linkage disequilibrium was more extensive in the putative source regions of Iberia and central Asia than in central Europe. Furthermore, although some population-genetic studies in *A. thaliana* have identified relatively unstructured patterns of genetic variation compatible with rapid range expansions from glacial refugia [17]–[20], the most recent studies of large data sets have found that genetic variation in *A. thaliana* shows evidence of considerable population structure 16,21,22. This structure has not been extensively analyzed to determine the likely explanations for its origin, and hypotheses about the location of origin and the timing of the spread of *A. thaliana* have been under some debate [20],[23],[24].

In this article, we consider an alternative model for the spread of *A. thaliana* in Europe. Using recently developed approximate Bayesian computation and spatial modeling techniques, we re-analyzed the data of Nordborg et al. [21], one of the largest population-genetic data sets collected to date in *A. thaliana*. We find evidence that a migration wave from east to west is responsible for most of the genetic ancestry of European *A. thaliana*. We discuss this result in relation to the hypothesis of an eastern refugium, and in relation to the hypothesis that the migration of *A. thaliana* may have been precipitated by the spread of agriculture into Europe.

## Results

### Population Structure, Clines, and Clusters

To investigate spatial population structure in European accessions of *Arabidopsis thaliana*, we used model-based clustering as implemented in the TESS computer program [25],[26]. Our analysis used the molecular data from 75 European accessions plus one accession from Libya (Mt-0), a total set of 876 alignments described in the study of Nordborg et al. [21] (Table S1). Using TESS, we performed an admixture analysis incorporating individual spatial coordinates and accounting for natural obstacles (see Methods and Figure S1). The program allows individuals to be distributed over *K*_max_ clusters, estimating the most likely value for the number of clusters as a value *K* less than or equal to *K*_max_ (see Methods).

**Figure 1:**
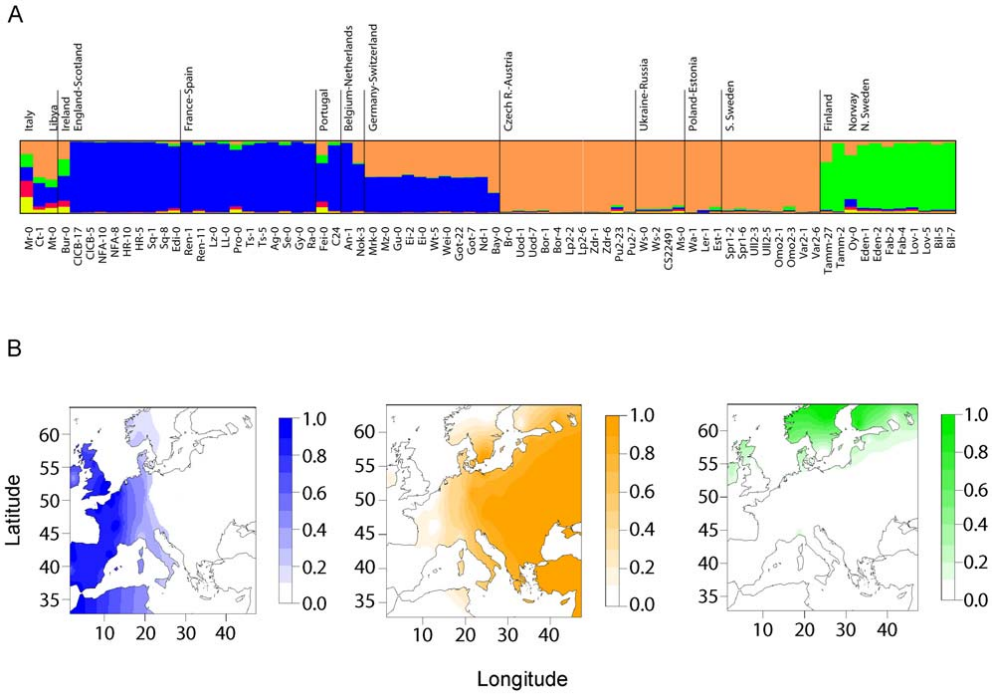
Bayesian clustering. (A) Membership coefficients in *K*_max_ = 5 putative populations, computed using the average values over the 10 TESS runs with the smallest values of the deviance information criterion from a total of 100 runs. Similar results were obtained with other values of *K*_max_ from 4 to 10. (B) Interpolated membership coefficients in the three apparent subpopulations: western cluster, eastern cluster, and northern cluster.

The TESS runs with the smallest values of the Deviance Information Criterion, a penalized measure of how well the model underlying TESS fits the data, were obtained for *K*_max_ greater than four (see Methods). In Figure 1, we report results for *K*_max_ = 5 clusters. The cluster membership coefficients estimated for the central European and western European accessions suggest that clinal variation occurs along an east-west gradient separating two clusters. The western cluster grouped accessions mainly from the British Isles, France and Iberia. The eastern cluster grouped all accessions from central Europe, southern Sweden, Poland, Russia, Ukraine, and Estonia. German and Swiss accessions shared almost the same amount of membership in the western and eastern clusters. The eight northern Swedish accessions and two Finnish accessions were grouped into a separate cluster.

In previous analysis of the same data set [21], it was observed that when individual genomes were clustered by genetic similarity using the program STRUCTURE [27], European accessions sorted into *K* = 8 clusters, some of them corresponding to small geographic regions [21]. The TESS analysis identified a substantially lower number of actual clusters (Figure 1), consistent with more continuous allele frequency variation across geographic space and with significant isolation by distance [15],[16],[22]. Although the northern European cluster was also identified from STRUCTURE runs with *K* = 3 [21], some differences were found by TESS in the two continental clusters. In [21], the Iberian accessions clustered with the eastern populations, whereas TESS grouped them with the western accessions (France, British Isles). More strongly than in the STRUCTURE analysis, the TESS results suggest clinal variation of allele frequencies within central and western Europe, with Germany possibly serving as a hybrid zone separating the two clusters corresponding to these regions.

### Orientation of the Cline

To better evaluate the direction of variation in the continental cluster, we regressed heterozygosity on geographic distance. This analysis used the approach of Ramachandran et al. [28], who showed that recurrent founder events can cause a decrease in genetic diversity in colonizing populations. Assuming a unique origin, genetic diversity is then predicted to decrease approximately linearly with geographic distance from the origin.

All accessions from the northern Sweden sample, as well as a few accessions that were poorly geographically connected to other accessions, were removed from the regression analysis. The remaining accessions were grouped into seven samples (Table S2), defined on the basis of geographic and genetic proximity. To minimize the sensitivity of the regression analysis to a particular geographic pooling of European accessions, we repeated the regression study for several combinations of seven modified samples, and the results reported can be viewed as representative of these various combinations.

**Figure 2:**
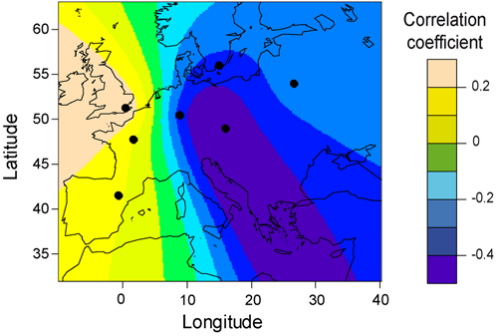
Diversity regressed on geographic distance. Correlation (*R*) map for the linear regression of expected heterozygosity on great circle distance. We used 300×180 points on a two-dimensional lattice covering Europe, and we computed distances from each lattice point considered as a potential source. The dots represent the centers of the 7 population samples used in the regression analysis.

For each of 300×180 points on a two-dimensional lattice covering Europe, we computed distances from each lattice point considered as a potential source for the geographic expansion of *A. thaliana*. The Pearson correlation coefficients of genetic diversity with distance from the source were estimated and plotted on the grid. The correlations were negative (∼ −0.5) in the east, and they were positive (∼ +0.3) in southwestern Europe. Assuming a unique site of origin, Figure 2 provides evidence that the pattern of heterozygosities is best explained by spatial expansion originating from the east.

Because this analysis is based on a relatively limited geographic sample, it is possible that it is affected by the peculiarities of this sample. Therefore, to assess the possibility of bias due to non-uniform and sparse geographic sampling, we performed spatially explicit range expansion simulations that reproduced the geographic sampling scheme of the actual data (Text S1). Assuming an origin in Anatolia (west Asia), we indeed observed a considerable shift of the position of the estimated origin to the southwest of the true origin (Figure S2). Because our data analysis identified a best-fitting origin in the Balkan region, it is thus possible that the true origin is potentially localized farther to the northeast.

### Statistical Evaluation of Alternative Models of Demographic Expansion

Inference of demographic parameters and the choice of a best-fitting demographic model for the data were performed using an approximate Bayesian computation (ABC) analysis [29]–[31]. ABC approaches bypass the computational difficulties of using explicit likelihood functions by simulating data from a coalescent model. These methods rely on the simulation of large numbers of data sets using parameter values sampled from prior distributions. A set of summary statistics is then calculated for each simulated sample, and each set of summaries is compared with the values for the observed sample, *s*_obs_. Parameter values that have generated summary statistics close enough to those of the observed data are retained to form an approximate sample from the posterior distribution, enabling parameter estimation and model choice (see Methods).

The ABC analysis was limited to a subset of 64 individuals representing the central European and western European populations. We restricted the analysis to the non-coding part of the genomic data, using the intron and the intergenic sequences only (648 loci). Simulated data also included 648 corresponding loci, each paired to have the same length as a locus in the observed data. The loci were assumed to be in linkage equilibrium, in agreement with the median ∼100 kb distance between fragments in the genome-wide data [21] and with levels of linkage disequilibrium that decay within ∼10 kb in *A. thaliana* [21],[32].

Coalescent simulations were performed under four demographic scenarios (Models A–D). Model A has a constant population size, *N*_0_. Model B has an exponentially growing population size (present size, *N*_0_, ancestral size, *N*_1_, time since the onset of expansion, *t*_0_). In model C, the population size was constant in the distant past as well as in the recent past, and the growth was exponential between the two periods of constant population size (present size, *N*_0_, ancestral size, *N*_1_, time since the onset of expansion, *t*_0_, time since the end of expansion, *t*_1_). Model D is similar to model B, but it includes an ancient bottleneck before expansion. The prior distributions used in the four models are described in Table S3. Twelve summary statistics were used to capture genomic information at the 648 loci (see Methods). To make quantitative model comparison possible, we evaluated the evidence of model 1 against model 2 (where 1 and 2 are chosen among A, B, C and D) using an approximation of the Bayes factor [33]. Pritchard et al. [30] computed the Bayes factor as the ratio of the acceptance rates in Models 1 and 2. Including smooth weighting to more heavily weight the simulations that produced results that more closely matched the observed data [29], we approximated the Bayes factor as
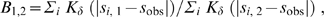
where *K_δ_* is the Epanechnikov kernel and *s_i_*_,1_ and *s_i_*_,2_ are the *i* ^th^ vectors of summary statistics simulated under models 1 and 2 (see Methods).

**Figure 3:**
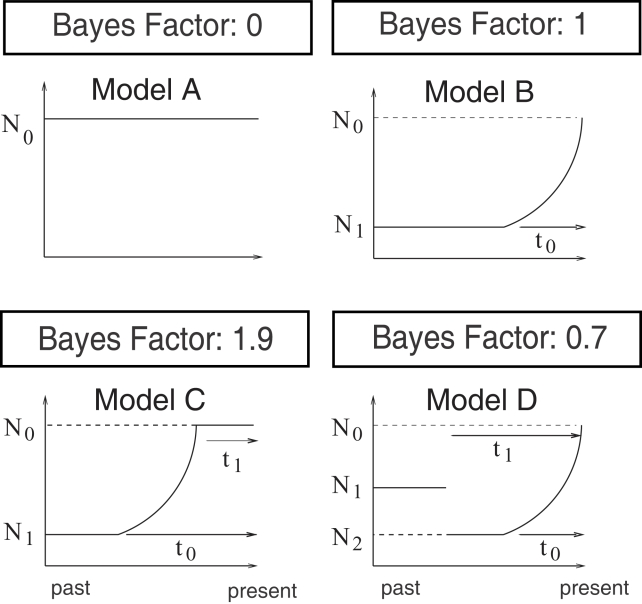
Bayes factors. The 4 demographic scenarios (Models A–D) and their associated Bayes factors. Model A is the model with constant population size, *N*_0_. Model B is a model with an exponentially growing population size (present size, *N*_0_, ancestral size, *N*_1_, time since the onset of expansion, *t*_0_). In Model C, the growth is exponential between two periods with constant size (present size, *N*_0_, ancestral size, *N*_1_, time since the onset of expansion, *t*_0_, time since the end of expansion, *t*_1_). Model D is similar to Model B, but it includes an ancient bottleneck before expansion. Variants of these 4 models, including variable mutation rates across loci, are considered here. The Bayes factors (top boxes) correspond to the ratio of the weight of evidence of each model to the weight of evidence of Model B. Two window sizes, *δ*_0.01_ and *δ*_0.05_, were used when computing the Bayes factors. These window sizes correspond to the 1% and 5% quantiles of the distance between the values of the summary statistics obtained under Model B and the observed values of the summary statistics. The Bayes factors were identical for the 2 window sizes and for values rounded for one decimal place, except for Model C, for which a minor difference was observed (1.8 for *δ*_0.05_ instead of 1.9).

Among all the scenarios, variants of the four models with variable mutation rates across loci were given higher statistical support, measured by the Bayes factor, than were models with fixed mutation rates - reflecting the high heterogeneity of diversity estimates among loci [21]. The best-supported model was model C with variable mutation rates, which assumed a past rapid expansion followed by a constant-size population phase (see Figure 3). The Bayes factor *B*_A,B_ = 0 indicates that the model with constant population size (model A) was totally unsupported. The exponential growth model (model B) was the second best-supported model, and the evidence supporting model C against model B was moderate (*B*_C,B_ = 1.9, see Figure 3). The scenario in which the population experienced a bottleneck before expansion was rejected, but less decisively than model A (model D, *B*_D,B_ = 0.7).

**Figure 4:**
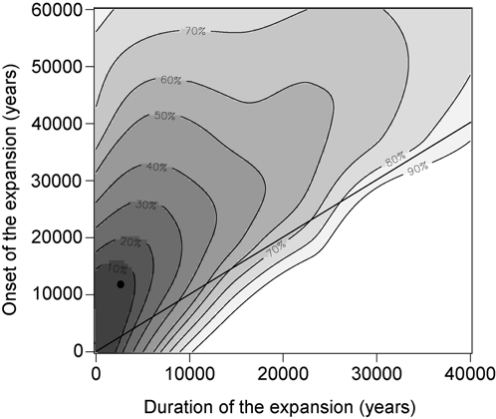
Onset and duration of the demographic expansion. Plot of the joint posterior distribution for the time of onset of the expansion, *t*_0_, and the length of the expansion, *t*_0_−*t*_1_. Computations were performed under demographic Model C, in which the population was initially constant, then grew exponentially until *t*_1_, and then remained constant until the present. Percentages represent the cumulative probabilities under the density curve. The straight line indicates that the duration of expansion cannot be longer than the time elapsed since the onset of expansion.

Table 1 displays the estimates of the parameter values under the variants of model B and C with variable mutation rates. The time of onset of the expansion was dated at *t*_0_ = 10,000 BP (model B) and *t*_0_ = 12,000 BP (model C) using the Maximum A Posteriori (MAP) estimate (Figure S3). As a consequence of using broad prior bounds in the ABC analysis, similarly to [34], we observed large 95% credibility intervals. The ratio of the ancestral population size to the present population size was estimated at *N*_1_/*N*_0_ = 0.3, but the large credibility interval (0,0.6) makes it impossible to eliminate the hypothesis of a wider expansion. The MAP estimate of the mutation rate was *μ* = 2.0×10^−8^ with credibility intervals ranging from 0.9×10^−8^ to 12.6×10^−8^. The MAP estimate for the date of the end of the expansion was *t*_1_ = 5,000 BP (see Table S4 and Table S5 for posterior estimates and Bayes factors for all eight models). To investigate the relationship between the time of onset, *t*_0_, and the length of the expansion, *t*_0_−*t*_1_, the joint posterior distribution of these two quantities was computed. Figure 4 displays this joint distribution, and it indicates a positive correlation between the two values.

**Table 1:** Estimates and 95% credibility intervals of parameter values under the variants of models B and C with variable mutation rates.

Model Parameters	Model B	Model C
μ (×10^−8^)	**2.0** (0.9,12.6)	**2.2** (1.1, 11.9)
*N_0_*	**179,000** (65, 1808)	**137,000** (72, 1228)
*t_0_*	**10,000** (4, 108)	**12,000** (5, 117)
*N_1_*	**76,000** (9, 474)	**59,000** (0, 447)
*N_0_/N_1_*	**0.3** (0.1, 0.6)	**0.3** (0, 0.6)
*t_1_*	-	**5,000** (0, 80)

The set of parameters included the mutation rate per bp per generation, *μ*, the present equilibrium population size, *N*_0_, the time since the onset of expansion, *t*_0_ (in years), the population size at the onset of expansion, *N*_1_, and the time elapsed since the equilibrium phase, *t*_1_ (in years). For each model, the 95% credibility interval of each parameter (×10^3^ for population sizes and times) is given after its maximum a posteriori estimate.

### Divergence Time and Migration Rate of Northern European and Central European Populations

**Figure 5:**
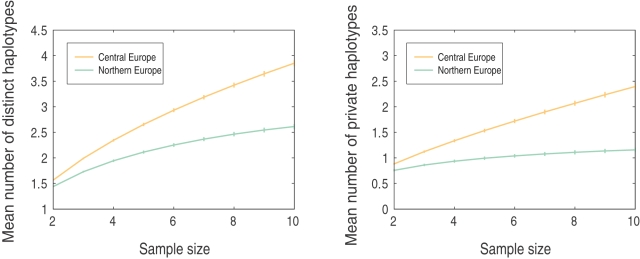
Number of distinct and private haplotypes. The mean number of distinct haplotypes and the mean number of private haplotypes of the central European population and the northern European population as functions of sample size. Vertical bars show standard error.

Because we observed considerable difference in the TESS analysis between the northernmost accessions and the main European populations (Figure 1), we performed model fitting to assess various scenarios for the split of the northern cluster. Quantifying the genetic divergence between the central European population and the northern Swedish and Finnish population by the mean number of distinct haplotypes and the mean number of private haplotypes [35], we obtained estimates of these statistics for subsamples of size two to ten. The patterns of haplotype diversity in the central European and northern European populations were typical for pairs of separated populations in which one population has larger size than the other [36]. The central European population had, on average, 3.85 distinct haplotypes for a sample of ten individuals, and the northern European population had, on average, 2.61 distinct haplotypes for a sample of ten individuals. However, in each population, about half of the haplotypes were unique to the population (Figure 5), and the genetic variation in the northern European population was not a subset of that in the central European population.

**Figure 6:**
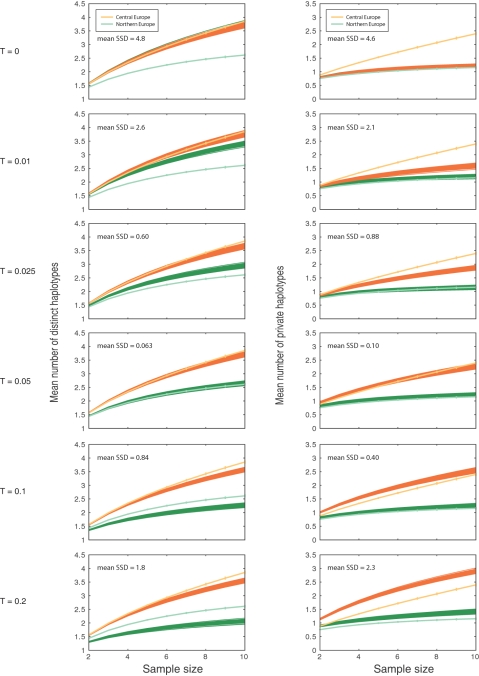
Estimation of the splitting time between the northern and central European populations of *A. thaliana*. The mean number of distinct haplotypes and the mean number of private haplotypes of two simulated populations, as functions of sample size. The dark orange lines show the simulation results for a population of size 135,000, and the dark green lines show the simulation results for a population of size 135,000×1/4. The top panel shows the case when the split time is 0. Below follow the results for increasing split times. No migration is assumed. The split time *T* is given in units of population size. The fit of the simulated data to the observed data was evaluated by the mean across the 100 simulations of the sum of squared differences (SSD) between each simulated data set and the observed data.

**Figure 7:**
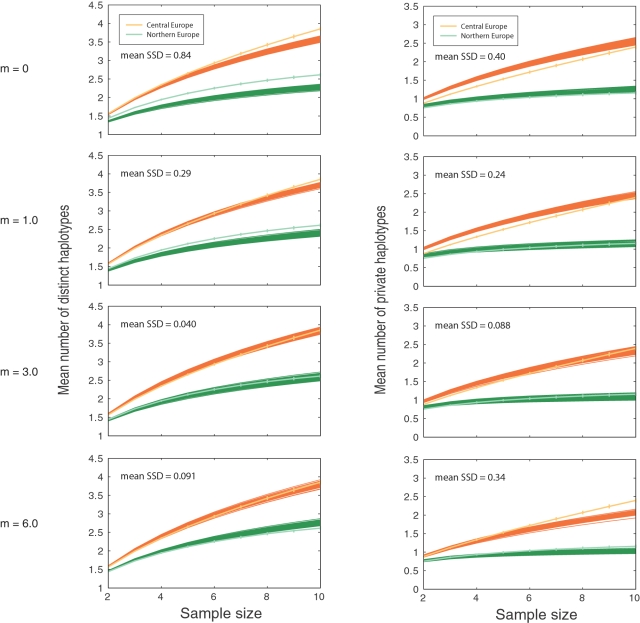
Estimation of the migration rate between the northern and central European populations of *A. thaliana*. The mean number of distinct haplotypes and the mean number of private haplotypes of two simulated populations as functions of sample size, shown for 100 replicates. The dark orange lines show the simulation results for a population of size N_CE_ = 135,000, and the dark green lines show the results for a population of size 135,000×1/4, when *T* = 13,500 years. The top panel shows the case when the migration rate, *m*, equals 0, and then follow the cases with *m* = 3 and *m* = 6 (normalized by *N*_CE_). The results from the observed populations are also plotted for comparison (lighter orange and green lines).

To study the split between the northern and central European populations, we used a coalescent model for the divergence between two populations at some time *T* in the past, with subsequent migration at rate *m* between these two populations (where *m* is the rate in each direction). We simulated the same number of fragments as in the data for both populations, and we determined the mean across 100 replicates of the sum of squared differences (SSD) between the simulated and the observed summary statistics. In a first set of simulations we increased the split time *T* from 0 to 135,000 years in a model with no migration (*m* = 0). Figure 6 shows the results for *T* = 0 to *T* = 27,000 BP superimposed on the same summary statistics computed for the observed data. For small values of *T*, the fit of the simulated data to the observed data was poor, with an improvement as *T* increased (SSD for *T* = 1,350 BP, distinct haplotypes: 2.56, private haplotypes: 2.12). When *T* was equal to 7,000 BP, the simulated data fit the observed data quite well (SSD for *T* = 7,000 BP, distinct haplotypes: 0.06, private haplotypes: 0.10). When *T* increased beyond 13,500 BP, the fit became poorer. In a second set of simulations, we used a population divergence model that incorporated migration, and we increased the values of the migration rate, *m*. Figure 7 shows simulated results superimposed on the observed results. The simulations fit the data relatively well for *m* in the range [1],[3] when *T* equalled 13,500 BP, and the best values were obtained for *m* = 3 (SSD for *m* = 3, distinct haplotypes: 0.04, private haplotypes: 0.08).

As *m* increased above the value 3, the fit of the mean number of distinct haplotypes deteriorated. We also tested values of *T*>13,500 together with *m*>3, without finding a close fit to the observed data, and the best fit was found for a model with low migration rates. A model with high migration rates was not able to replicate the observed data under the tested conditions. Thus, it is unlikely that the split occurred more recently than ∼7,000 years ago.

### Range Expansion and Spatial Simulations

In the ABC analysis the scenarios that consisted solely of population size change produced patterns of DNA sequence diversity similar to those resulting from a rapid spatial range expansion [37]. To better include geographic sampling in the analysis and to estimate the rate of spread, we modeled the process of colonization of Europe in a more explicit manner [38],[39]. Range expansion was simulated under a two-dimensional wave-of-advance model [40]. We included environmental heterogeneity, borrowing topographic information from a Geographic Information System. Assuming an origin of the colonization process to the north of the Black Sea (48°N, 35°E), we divided Europe into an array consisting of 130×180 = 23,400 demes, each representing an area of ∼2,500 km^2^. To account for the fact that in Europe, *A. thaliana* grows mainly in low-altitude landscapes, carrying capacities were set to their highest values for altitudes below 200 m and were linearly decreased for altitudes higher than 1,500 m.

It has been previously recognized that the frequency spectrum may be influenced by signals of past demographic events [41],[42]. Consequently, the fit of simulated data to the pattern of polymorphism of *A. thaliana* was evaluated by comparing the non-coding empirical folded frequency spectrum and frequency spectra obtained from simulated individuals located at the same coordinates as the real accessions. Simulated and observed frequency spectra were compared by using the *χ* ^2^ distance (see Methods).

A coarse preliminary search found that values of migration rates and growth rates corresponding to the saturation of a deme in 100–300 years and lengths of the colonization phase around 3,000–6,000 years followed by an equilibrium migration phase yielded non-significant *χ* ^2^ *P-*values. Thus, these values provide a reasonable explanation for the observed data. They translate into a wave-of-advance of around 0.5 to 1 km/year.

**Figure 8:**
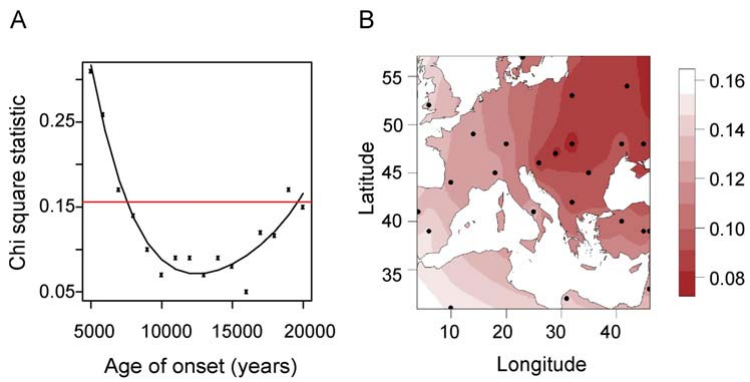
Chi-square statistic maps for spatial range expansion. (A) *χ* ^2^ distances between the simulated and the empirical folded frequency spectra as a function of the time of onset of the expansion. The other parameters were fixed at *m* = 0.25, *r* = 0.6–1.2, and *N*_1_ = 10,000. The origin was placed north of the Black Sea (48°N, 35°E). The horizontal line corresponds to the 95% rejection interval of the *χ* ^2^ test (df = 3, see Methods). (B) Interpolated map of *χ* ^2^ distances between simulated and empirical folded spectra for 24 potential origins (black dots). The time of onset was fixed at 9,000 years BP, and the other parameters were fixed as in (A).

In a second stage of the analysis, we investigated the time at which the range expansion began, varying this time from *t*_0_ = 5,000 BP to *t*_0_ = 20,000 BP assuming a growth rate of *r* = 0.6 for the oldest dates. For the most recent dates, we increased *r* to 0.7, 0.9 and 1.2 so that the colonization phase ended before the present day. This analysis supported the values found by the MAP estimate from the ABC analysis. Figure 8A shows that dates around 10,000–12,000 BP are consistent with the pattern of polymorphism observed today.

To better locate the origin of *A. thaliana*, we investigated several potential locations, and we plotted *χ* ^2^ distances between simulated spectra and the empirical spectrum on an interpolated map (Figure 8B). The *χ* ^2^ values ranged from 0.03 (East) to 0.3 (Spain - North Africa). Although the map does not provide an accurate localization of the onset of range expansion, it is similar to Figure 2, providing further support to the hypothesis of an eastern origin.

**Figure 9:**
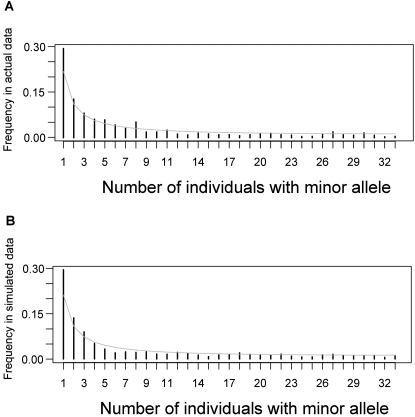
Frequency spectrum in actual and simulated data. Minor allele frequency spectra of empirical data and data simulated under the best-fitting model of spatial range expansion. Population growth followed the logistic model within each deme (see text for the other parameter settings). The solid line (grey) corresponds to the neutral folded frequency spectrum. (A) The empirical folded spectrum was computed from the 648 inter-genic and non-coding sequences. (B) The simulated spectrum was computed using the same number of neutral nucleotides as in the data. In simulations, expansion started 9,000 years ago from a potential origin north of the Black Sea (48°N, 35°E). Other locations from a large region around this potential origin yielded very similar simulated spectra.

Figure 9A demonstrates that the empirical folded frequency spectrum computed from non-coding nucleotides deviates from neutrality through an excess of rare alleles. Figure 9B shows one simulated folded spectrum obtained from the estimated parameters (*m* = 0.25, *r* = 0.6, *N*_1_ = 5,000 and *t*_0_ = 10,000, *χ* ^2^ = 0.03, *P* = 0.68). For this set of parameters, the estimated speed of the wave-of-advance was ∼0.9 km/year. It is clear from the search strategy used here that these parameter settings are only likely to represent a local maximum of the probability of an evolutionary scenario, and that other settings may also provide a reasonable fit to the data.

## Discussion

We have performed an investigation of the population structure and demographic history of European *A. thaliana*, using genome-wide sequence data collected in accessions from across Europe. Our main results are as follows. (1) On the basis of spatial Bayesian analysis with TESS, we observed that most European accessions were distributed over three clusters: one northern European cluster and an east-west cline of variation across continental Europe (Figure 1). (2) The level of genetic variation is greater in the east than in the west; if a single-origin model is used for modeling genetic diversity in European populations, the most likely source location is in the east and the estimated rate of westward spread is ∼0.9 km/year (Figures 2 and 8). (3) Simulations suggest that the pattern of genetic variation is explained most parsimoniously by an ancient split of the northern cluster from the central European cluster >7,000 BP. (4) Approximate Bayesian computation suggests that the European *A. thaliana* population began an expansion in size ∼10,000 BP, lasting 5,000 years (Figures 4 and 8).

### Natural Colonization After the Ice Age

From a biogeographic point of view, Europe is a large peninsula with an east-west orientation, delimited in the south by a strong Mediterranean barrier. During glaciation epochs, many species likely went through alternating contractions and expansions of range, involving extinctions of northern populations when the temperature decreased, and spread of the southern populations from different refugial areas after glaciation. Such colonization processes were likely characterized by recurrent bottlenecks that would have led to a loss of diversity in the northern populations.

The idea that the refugia were localized in three areas (Iberia, Italy, Balkans) is now well-established [12], although recent studies, particularly of tree species, have begun to suggest that northern and eastern refugia could have existed [43],[44]. Comparison of colonization routes has highlighted four main suture-zones where lineages from different refugia meet [11]. Two of these suture-zones correspond to the Alps and the Pyrenees, while the two others are in Germany and in Scandinavia.

We observed that genetically diverse populations of *A. thaliana* were localized at intermediate latitudes, as a potential consequence of the admixture of divergent lineages colonizing the continent from separate refugia. These results are potentially consistent with the pattern expected if the species colonized Europe from two separate refugia, one in the Iberian peninsula and the second in the east, as suggested by the model of Sharbel et al. [15]. Similarity with patterns of cpDNA diversity in 22 plant species that have genetically divergent populations in Mediterranean regions was also observed for the seven geographic samples considered in the regression analysis ([13] and Figure S4). Furthermore, the presence of a highly divergent accession (Mr-0) in Italy, south of the Alpine barrier, is also compatible with the view that *A. thaliana* was present in Mediterranean refugia during the last glaciation.

We observed that intraspecific diversity declines away from the southeast, as predicted by a model of successive founder events during colonization. We also inferred that the putative origin of most accessions in the sample is localized somewhere in a vast eastern region, encompassing refugia such as the Caucasus region and the Balkans. The direction of diffusion from the east towards the British Isles coincides with the post-glacial re-colonization of Europe for many species such as beech, alder and ash trees, or flightless grasshopers [45],[46], and it is possible that, to a large extent, this wave of expansion erased any contribution of ancient western lineages that originated in Mediterranean refugia.

### Colonization of Fennoscandia

The boreal regions, in which environmental conditions are often very severe, contain the northern distribution limit of many European plants. These regions are often characterized by larger fluctuations in population size, which increase the effect of drift and can lead to increased genetic differentiation [47]. Fennoscandia has recovered its flora after the last ice age, less than 10,000 years ago, via many different routes. The presence of a suture-zone in Scandinavia indicates that this area may have been colonized by *A. thaliana* both from the south and from the northeast. The estimated separation time of the northern European *A. thaliana* population and the central European population, at least 7,000 years ago, indicates that the split between the continental and northern populations took place during the early history of the re-colonization of Europe by the species.

### Ecological Imperialism in Europe

An alternative hypothesis to the idea of a natural spatial expansion of *A. thaliana* is that its spread might have accompanied the spread of farming into Europe, perhaps following an earlier post-glacial wave of colonization. Between 9,000 and 5,500 BP Neolithic farming spread across Europe from the Near East, primarily northwestwards along the Danube-Rhine axis [48]–[50]. Several aspects of our results are consistent with the hypothesis that *A. thaliana* was part of a group of weeds that accompanied the spread of agriculture into Europe. First, the evidence for an eastern source for European *A. thaliana* parallels the evidence that agriculture spread into Europe from the east [48],[49]. Putative origins in the Danube basin, west of the Black Sea, received high explanatory power in our analysis, and this area was an important way-point in the route followed by the spread of agriculture. Second, the estimated time for the beginning of the *A. thaliana* population size expansion parallels the time for the spread of agriculture. Third, the estimated rate of westward spread of *A. thaliana*, ∼0.9 km/year, fits within the range 0.6–1.3 km/year estimated for the rate of agricultural expansion [48],[51]. It is believed that Neolithic agriculture advanced into Europe along two preferred routes, a Mediterranean route and a Danubian route [52],[53]; our analysis suggests that if *A. thaliana* followed the spread of agriculture, then it likely followed the Danubian route.

The possible prehistoric anthropogenic spread of *A. thaliana* in Europe is an instance of a more general pattern documented in historical times, in which land disturbances instigated through long-distance human migrations co-occur with the spread of opportunistic organisms unintentionally brought by the migrating populations from their home region. This phenomenon of “ecological imperialism” has been used to explain the current prominence of European weeds in regions of the Americas, Australia, and New Zealand that have recently been transformed by European agriculture [54]. Several lines of evidence support the view that a similar process for the spread of weeds acted during the transformation of European landscapes by the spatial advance of agriculture - that is, that a large fraction of weeds in Europe trace their geographic distributions to the Neolithic expansion of European farming. For example, based on palaeobotanical data, Pyšek et al. [55] estimated that of the presently known prehistoric alien species of central Europe, 35% arrived there during the first thousand years after the advent of agriculture. Kreuz et al. [56] detected a chronological correlation in the number of introduced weed species in central Europe and the development of the agriculturalist Bandkeramik culture. In two weedy species of *Lolium*, Balfourier et al. [57] found patterns of population structure explicable by the spread of agriculture, supporting the view that the *A. thaliana* results could be part of a general trend for prehistoric European weeds. Another source of evidence for a large-scale prehistoric agriculturalist spread of weeds into Europe is a comparison of weed species in modern plots of land in the Czech Republic. In the study of Pyšek et al. [58], introduced weeds that entered Europe in prehistoric times were comparatively more numerous in land farmed with crops dating to the origin of European agriculture (e.g. barley and wheat) than in land farmed with more recently introduced crops (e.g. maize and rapeseed), where recently introduced weeds were more numerous. Thus, the success in modern times of *A. thaliana* and other weedy plants brought from Europe to temperate regions worldwide may be the result of long-lasting associations with European agriculture that these plants have had since the time of the Neolithic revolution.

While our results might be explained by the simultaneous expansion of *A. thaliana* into Europe from multiple glacial refugia, we find that a perspective incorporating agriculture explains the data as parsimoniously as a model relying exclusively on natural dispersal. Because the sampling of accessions was denser at intermediate latitudes than it was in southern Europe, we were not able to exclude roles for Spanish or Italian refugia or for a Mediterranean route of agriculture in producing the pattern of variation in current genomes. One possibility is that *A. thaliana* did follow the agricultural expansion, but only after it had already arrived in Europe via a natural colonization from glacial refugia. Similarly to the diffusion of human agriculturalist genes, the continuous pattern of variation in *A. thaliana* would then be explained by the genetic dilution of the eastern genes that might have resulted from admixture with local populations during the agricultural expansion phase. Although the current data set has a large representation of individuals along the Danubian route of agricultural expansion, genomic analysis of a larger sample from Spain and the Balkans, as well as from the key eastern region of Asia Minor, will have greater potential to distinguish among possible models for the evolutionary history of *A. thaliana* in Europe.

## Methods

### Data Description

A set of 76 individuals containing both hierarchical population samples and stock center accessions was extracted from the sample of 96 individuals studied by Nordborg et al. [21]. The subset included all accessions within an interval of latitudes of (32°N, 65°N) and within an interval of longitudes of (−10°E, 40°E), i.e. all European accessions plus one from Libya (Mt-0). For the 76 individuals, the total set of 876 reliable alignments representing 0.48 Mb of the genome was used. A thorough description of the data set can be found in the Materials and Methods of [21]. The list of accessions used in this study can be found in Table S1.

### Spatial Population Structure

Since *Arabidopsis thaliana* is largely homozygous, we used a haploid setting. To enable comparisons with results obtained in [21] from the program STRUCTURE version 2.0, each fragment was treated as a multiallelic locus, so that two accessions had a different allele if they differed at any site in the fragment. To determine which clusters are generally robust to the assumption of continuous variation, we used a modified algorithm that includes spatially explicit prior distributions describing which sets of individuals are likely to have similar cluster membership [25]. In this approach, implemented in the program TESS [26], clusters correspond to spatially and genetically continuous units separated by small discontinuities that occur where genetic barriers are crossed. The incorporation of a spatial component into the clustering model has the potential to determine if clines provide a sensible description of the underlying pattern of variation.

We performed an admixture analysis using TESS version 1.1, whose individual-based spatially explicit Bayesian clustering algorithm uses a hidden Markov random field model to compute the proportion of individual genomes originating in *K* populations [25],[26]. The hidden Markov random field accounts for spatial connectivities by representing them as links in a network of individuals. In addition, the hidden Markov random field also incorporates decay of membership coefficient correlation with distance (computed on the network), a property similar to isolation-by-distance. The network topology merely conveys information about which pairs of individual genomes are more likely to be assigned to the same clusters, and the network was automatically generated by the TESS program using a Dirichlet tessellation obtained from the accession spatial coordinates. To better account for potential geographic barriers, we modified the network by removing several links. For our application to *A. thaliana*, we imposed a network topology in which the skeleton of the topographic structure of European landmasses was mimicked (Figure S1). This topology was obtained after removing the longest Dirichlet edges in the automatically generated graph.

Two values of the TESS interaction parameter were used, *ψ* = 0.6 and *ψ* = 1, which can be viewed as a moderate and a strong value. This hyperprior parameter weights the relative importance given to spatial connectivities (the value *ψ* = 0 recovers the model underlying STRUCTURE). Similar results were obtained from both the moderate and strong values, and only those for *ψ* = 0.6 are reported.

TESS and STRUCTURE proceed with the determination of the number of clusters *K* in a similar way. However the TESS algorithm incorporates a regularization procedure that perhaps leads to a less ambiguous decision regarding *K*. Indeed *K* can be determined by sequentially increasing the maximal number of clusters, *K*_max_, and by running the program until the final inferred number of clusters, *K*, becomes less than *K*_max_. We used the admixture version of TESS, and we set the admixture parameter to α = 1. The algorithm was run with a burn-in period of length 20,000 cycles, and estimation was performed using 30,000 additional cycles. We increased the maximal number of clusters from *K*_max_ = 3 to *K*_max_ = 8 (20 replicates for each value). Runs with *K*_max_ = 5 led to either *K* = 3 or to *K* = 4.

For each run we computed the Deviance Information Criterion (DIC) [59], a model-complexity penalized measure of how well the model fits the data. The smallest DIC values were obtained for *K*_max_ = 5. One accession, Mr-0 (Italy), shared nearly equal membership in each of the *K*_max_ clusters, regardless of the value of *K*_max_ (see the clustering tree in [60] for identification of Mr-0 as an outgroup accession). To a lesser extent, Bur-0 (Ireland) and Fei-0 (Portugal) exhibited similar patterns of shared membership. For *K*_max_ = 5, we performed 100 additional runs (interaction parameter *ψ* = 0.6, admixture parameter α = 1), and we averaged the estimated admixture coefficients (Q matrix) over the ten runs with the smallest values of the DIC (DIC ∼72,000, s.d. = 30). To account for label switching and to decide which of the clusters of each run corresponded to a specific label, we used the software CLUMPP version 1.1 [61], whose greedy algorithm computed a symmetric similarity coefficient equal to 0.788 (100 random input sequences, *G* statistic).

### Spatial Interpolation

Spatial interpolation of admixture coefficients was performed according to the kriging method as implemented in the R packages ‘spatial’ and ‘fields’ [62],[63]. One difficulty with fitting trend surfaces arises when the observations are not regularly spaced. To handle this issue we took the spatial correlation of the fitting errors into account by assuming that the errors had non-null covariance. Trend surfaces of degree two were adjusted using generalized least squares and exponential covariance with decay parameter *h* = 5.

### Heterozygosities Regressed on Geographic Distances

The regression analysis of heterozygosities on geographic distances was based on 57 central European, eastern and western European accessions. The 57 individuals were grouped into seven samples as described in Table S2. The seven samples were defined on the basis of geographic and genetic proximity, and provided a balance between pooled individual accessions and actual population samples. We did not include nine individuals that were either ambiguously assigned to clusters by TESS or that were geographically isolated. The German sample was restricted to six accessions, and diversity for this sample was estimated by using a resampling procedure (mean over 100 replicates). We also ran a simulation study to evaluate the influence of the resampling strategy (Text S1).

### Approximate Bayesian Inference for Demographic Scenarios

We used an ABC approach for inferring demographic parameters under four models of population growth. In the ABC approach, we assume that there is a multidimensional parameter of interest *θ*, and the observed value *s*_obs_ of a set of summary statistics, *S*, is calculated for the data. The basic rejection sampling method generates random draws (*θ_i_, s_i_*), where *θ_i_* is sampled from the prior distribution, and *s_i_* is measured from synthetic data, simulated from a generative model with parameter *θ_i_*. Fixing the tolerance error, *δ*, only parameters *θ_i_* such that |*s*_obs_−*s_i_*|<*δ* are retained to form an approximate sample of size *M* from the posterior distribution, where | ^.^ | is the Euclidean norm. We used tolerance errors such that fractions of either 5% or 1% of the total number of simulations were retained.

The four demographic scenarios were described in the text as Models A–D. The six-dimensional parameter *θ* included the mutation rate per bp per generation, *μ* (×10^−8^), the population size at the onset of expansion, *N*_1_, the time since the onset of expansion, *t*_0_, the growth rate, *r*, the present equilibrium population size, *N*_0_, and the time elapsed since the equilibrium phase, *t*_1_. The variable mutation rate models included locus-specic rates, *μ_j_*_,_ obtained as independent realizations of an exponential prior distribution for which the hyperparameter was exponentially distributed with mean *μ*. Coalescent simulations were performed with the software MS [64]. Recombination within each locus was assumed, using an exponentially distributed prior of mean 0.3 for the effective recombination rate [65]. The prior distributions used in the four models are described in Table S3.

Twelve summary statistics were used to capture genomic information at the 648 loci, defined as the 25%, 50% and 75% quantiles (quartiles) of each of the distributions of the number of segregating sites, the mean number of pairwise differences between sequences, the Tajima *D* statistic, and the number of distinct haplotypes. The summary statistics were rescaled before comparison to the observed statistics. We divided each simulated summary statistic by the median absolute deviation – a robust estimate of the standard deviation – of the simulated statistics. Our ABC approach partially followed Beaumont et al. [29], who added regression adjustment and smooth weighting to the Bayesian rejection algorithm of Pritchard et al. [30]. We dropped the regression adjustment step because it led to a poor fit during preliminary runs (*R* ^2^<0.25). The second improvement of the original method – namely, smooth weighting – was retained in our analysis. Smoothing was implemented using the Epanechnikov kernel *K_δ_* with window size *δ* to weight the parameters by *K_δ_* (|*s_i_*−*s*_obs_|) [29]. The same weights were also used when estimating the mean, the quartiles and the maximum of the posterior distribution.

We computed the Bayes factor when evaluating the evidence of model 1 against model 2 (where 1 and 2 are chosen among A, B, C and D) as described in Results. The new formula can be seen as an improvement of the method that used the ratio of acceptances under the two models to approximate the Bayes factor, originally formulated as
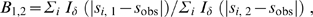
where *I_δ_* is the indicator function *I_δ_ (t)* = 1 if *t<δ*, 0 otherwise. Note that in our case, Jeffreys' scale on degrees of belief should be interpreted more cautiously than the usual scale based on exact Bayesian computation [33]. The Bayes factors in Figure 3 and Table S5 correspond to the ratio of the weight of evidence of each model to the weight of evidence of the variant of model B with variable mutation rates. Two tolerance errors, *δ*_0.01_ and *δ*_0.05_, corresponding to the 1% and 5% quantiles of the distance between the summary statistics obtained under the variant of model B with variable mutation rates and the observed summary statistics, were used when computing the Bayes factors.

### Divergence Time and Migration Rate of Northern European and Central European Populations

We selected 64 individuals from central Europe and western Europe and ten individuals from northern Europe (northern Sweden and Finland). From the 876 fragments, we removed indels, sites with more than 20% missing data, and monomorphic sites. A total of 795 fragments and 11,134 SNPs remained. For each site, the remaining missing data was replaced by sampling alleles from the allele frequency distribution so that the final data set did not contain any missing data.

We simulated data from model C using MS [64]. Forward in time, there is a period of constant population size followed by a period of growth and finally a period of constant population size ending in the present. We used the model parameters from the MAP estimates of model C (see Table 1), which received the most statistical support from the ABC analysis. We considered variable mutation rates per simulated fragment, taken from the same exponential distribution as used in the ABC analysis (also in agreement with [66]). The recombination rate in a simulated fragment was set to 0.3 [65]. We assumed that the population split into two subpopulations some time *T* in the past, scaled by *N*_CE_ = 135,000, the estimated size of the central European population, and that migration occurred at rate *m*, scaled by *N*_CE_. The size of the northern population, *N*_NE_ , was assumed to be 1/4 of the estimated size of the central European population, *N*_CE_. The growth scenario was assumed to be the same in the two populations, with only the population sizes differing.

To approximate the likelihood of the parameters, we used two haplotype diversity statistics, the mean number of distinct haplotypes and the mean number of private haplotypes. To correct the number of distinct haplotypes and the number of private haplotypes statistics for sample size differences, we used the rarefaction method [35],[36] to get estimates of these statistics for samples of size two to ten (the sample size of the northern Swedish and Finnish population was equal to ten). This was done separately for each fragment, and finally we took the average across fragments.

### Range Expansion and Spatially Explicit Simulations

Simulations of a two-dimensional stepping stone model were performed using the program SPLATCHE 1.1 [40]. We modeled Europe using an array of demes that included topographic information borrowed from the online Geographic Information System GEODAS of the National Geographic Data Center. The map covered latitudes from 32°N to 65°N and longitudes in an interval of −10°E to 40°E. Topography was used to define carrying capacities for each deme. We divided Europe into an array consisting of 130×180 = 23,400 demes, each representing an area of ∼600 km^2^. To account for the fact that *A. thaliana* inhabits lower altitude landscapes, carrying capacities were set to their highest values for altitudes below 200 m (*N* = 5,000). They were progressively decreased to *N* = 100 for altitudes higher than 1500 m using a nonincreasing step function (*N* = 2,500 for altitudes between 200 m and 500 m, *N* = 1,000 for altitudes between 500 m and 1000 m, *N* = 500 for altitudes between 1000 m and 1500 m). At the beginning of the colonization process, a single deme was occupied. To date the onset of the spread, we based the origin at the north of the Black Sea (48°N, 35°E). We chose a logistic population growth model to describe the dynamics of population demography within each deme. The growth rate *r* was identical in each deme. Following [66] we set the mutation rate per base pair and per generation around *u* ∼10^−8^, and the generation time corresponded to one year. Because the memory requirements of SPLATCHE are particularly high, we modified the mutation rate and the effective size in order to accelerate the generation time from one year to ten years (this means that the model was simulated ten generations at a time).

Values of the original population size were taken equal to *N*_1_ around 10,000 (5,000–15,000). DNA sequences were simulated using the modified mutation rate *v* = 10^−5^. Rescaling the generation time to a value *t*_R_ *= *10 years produced a level of nucleotide diversity close to the one present in the data (*N*_e_ *u* = *N*_1_×*v/t*_R_ ∼10^−2^). Note that *N*_1_ cannot be compared to the value used in the non-spatial ABC simulations unless we restore the original mutation rates and generation times. After the correction, the values used in the spatial and non-spatial scenarios were actually similar. In simulations, we assumed that the population remained constant (equal to *N*_1_) during 100 Ky before range expansion.

To compare with the data in western and central Europe, we simulated the genealogies of 66 individuals located at the same spatial coordinates as the set of 66 accessions that excluded those from northern Sweden and Finland. The fit of simulated data to the real data was assessed by evaluating the distance between the empirical folded frequency spectrum computed from the non-coding sequences, and frequency spectra obtained from individuals simulated at the same locations.

The distance used to compare folded spectra was the *χ* ^2^ distance defined from four classes as follows: Class 1) minor allele frequency 1 (total 28%); Class 2) minor allele frequency 2–4 (total 26%); Class 3) minor allele frequency 5–12 (total 25%); and Class 4) minor allele frequency 13–33 (total 21%).

Five model parameters were varied: the time of the onset of spatial expansion t0, the migration rate *m*, the growth rate within a newly colonized deme *r*, the effective population size at the beginning of range expansion *N*_1_ (resized), and the location of the origin. Ideally one would use an ABC analysis to choose a subset of parameters that maximizes the posterior probability of the corresponding evolutionary scenario given prior distributions over these parameters. However performing an ABC analysis with geographically explicit simulations is prohibitively time-consuming, due to the large cost of a single simulation. In practice, we first performed a coarse search using fixed values of the starting date t0 (equal to 8,000–12,000 BP) and a random sampling design for the other parameters, exploring migration rates (*m*) within the range 0.1–0.8 and population size expansion rates (*r*) within the range 0.2–1.5, and assuming that the starting point was located at coordinates (48°N, 35°E). This preliminary search found that values of migration rates around 0.2–0.3, growth rates between 0.6 and 1, and initial sizes of 5,000–10,000 individuals yielded non-significant *χ* ^2^ *P-*values. These ranges of parameter settings for *m* and *r* corresponded to the saturation of a deme in 100–300 years. For most of the simulations, the length of the colonization phase was around 3,000–6,000 years, which corresponded to waves of advance varying from 0.5 to 1 km/year. In a second stage, we investigated the time at which the range expansion began, varying this time from *t*_0_ = 5,000 BP to *t*_0_ = 20,000 BP using *r* = 0.6 for the oldest dates. For the most recent dates, we increased *r* to 0.7 (*t*_0_ = 10,000), 0.9 (*t*_0_ = 7,000) and 1.2 (*t*_0_ = 5,000), so that the colonization phase ended before the present day. Finally, we studied the explanatory power of twenty-four potential spatial origins throughout central and western Europe (*m* = 0.25, *r* = 0.6, Figure 8B).

## Supporting Information

Figure S1 **The skeleton of Europe.** The TESS hidden Markov model relies on a graph that specifies which pairs of individuals are most likely to be assigned to the same cluster. In this graph, the vertices correspond to the accessions, and the links represent their spatial connectivity.
(.07 MB PDF)

Figure S2 **Sensitivity of the regression analysis to the geographic sampling scheme.** The analysis was based on geographically explicit simulations using the computer program SPLATCHE. We assumed a date of onset of spatial expansion 10,000 years ago, carrying capacities in the interval (100, 5,000), migration rate *m* = 0.25, and growth rate *r* = 0.6. An Anatolian origin for the expansion was assumed, the origin was located at latitude 38°N and longitude 38°E, and was represented by a cross symbol in the figure. We generated 10 replicates of the simulation scenario, and, for each simulated data set, we inferred the most probable location for a putative origin by optimizing the *R* ^2^ statistic calculated in the regression of diversity on distance to the putative origin. The sampling scheme was identical to the one used to collect the actual data. The sample barycenter locations were 1: Southern Sweden, 2: British Isles, 3: France-Belgium, 4: Germany, 5: Iberia, 6: Central Europe, 7: Northeastern Europe (Table S2). The large circle surrounds the positions of the ten inferred origins, and the black dot represents their average position. See Text S1 for a more detailed discussion.
(.04 MB PDF)

Figure S3 **Posterior distribution for the time** ***N*** **_0_ since the beginning of the expansion.** The red solid line corresponds to Model C, for which the population size was initially constant, then grew exponentially from time *t*_0_ to time *t*_1_, and was constant again until the present. The dashed blue line corresponds to model B, for which the population size was initially constant, and then grew exponentially until the present.
(.07 MB PDF)

Figure S4 **Mean number of distinct haplotypes in the seven samples used in the regression analysis.** Higher values are in black circles, lower values are in white circles, and circle diameter is proportional to the mean number of distinct haplotypes. Exact values: Southern Sweden: 2.80, British Isles: 2.59, France/Belgium: 2.72, Germany: 2.72, Iberia: 2.41, Central Europe: 3.30, Eastern Europe: 2.61. See Table S2 for a description of the samples.
(.02 MB PDF)

Table S1 **List of 76 accessions used in the study.** The geographic coordinates of Pu2-7 and Pu2-23 have been corrected to 49.42°N and 16.36°E (M. Nordborg, personal communication). See Nordborg et al. [21], Tables S1 and S2, for complete information about population samples and stock center accessions.
(.02 MB PDF)

Table S2 **List of 7 samples used in the regression analysis of diversity on great circle distance.** The samples were defined on the basis of geographic criteria. We corrected for the fact that the German sample contains twice the number of accessions present in France, Iberia, or eastern Europe by randomly sampling 6 accessions in this population, and we averaged heterozygosity over 100 replicates. The British Isles, Central Europe, and southern Sweden contain pre-defined populations consisting of more closely related individuals.
(.01 MB PDF)

Table S3 **Prior distributions of parameter values under the various demographic models used during the ABC analysis.** The parameter *N*_0_ is the present population size, *N*_1_ is the population size at the onset of expansion, *r* is the exponential growth rate (that is, the population size at time *t* before present is *N*(*t*) = *N*_0_ *e^−rt ^*), *t*_0_ is the time since the start of the expansion, and *t*_1_ is the time since population size reached an equilibrium value. Time is measured backwards and in coalescent units of *N*_0_ generations. LN denotes the log-normal distribution, and *Г* stands for the Gamma distribution.
(.21 MB PDF)

Table S4 **Posterior distributions in the ABC analysis.** Estimates of parameter values under four demographic models and their variants with variable mutation rates. For each parameter, the MAP estimate is followed by the 95% credibility interval.
(.11 MB PDF)

Table S5 **Bayes factors.** The Bayes factors correspond to the ratio of the weight of evidence of each model to the weight of evidence of the variant of Model B with variable mutation rates. Two window sizes (or tolerance errors), *δ*_0.01_ and *δ*_0.05_, were used when computing the Bayes factors. These window sizes correspond to the 1% and 5% quantiles of the distance between observed summary statistics and the summary statistics obtained under the variant of Model B with variable mutation rates.
(.02 MB PDF)

Text S1 **Supplementary text.**
(.08 MB PDF)
