# Supplementary figures and images for: Demographic History of European Populations of Arabidopsis thaliana
Source: PLoS Genet. 2008 May 16;4(5):e1000075. doi: 10.1371/journal.pgen.1000075 (PMC2364639; doi:10.1371/journal.pgen.1000075)

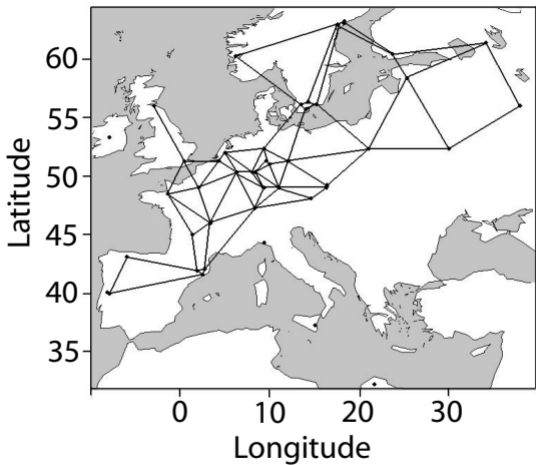

Supplement: Figure S1 — The skeleton of Europe. The TESS hidden Markov model relies on a graph that specifies which pairs of individuals are most likely to be assigned to the same cluster. In this graph, the vertices correspond to the accessions, and the links represent their spatial connectivity. (.07 MB PDF) [file pgen.1000075.s001.pdf]

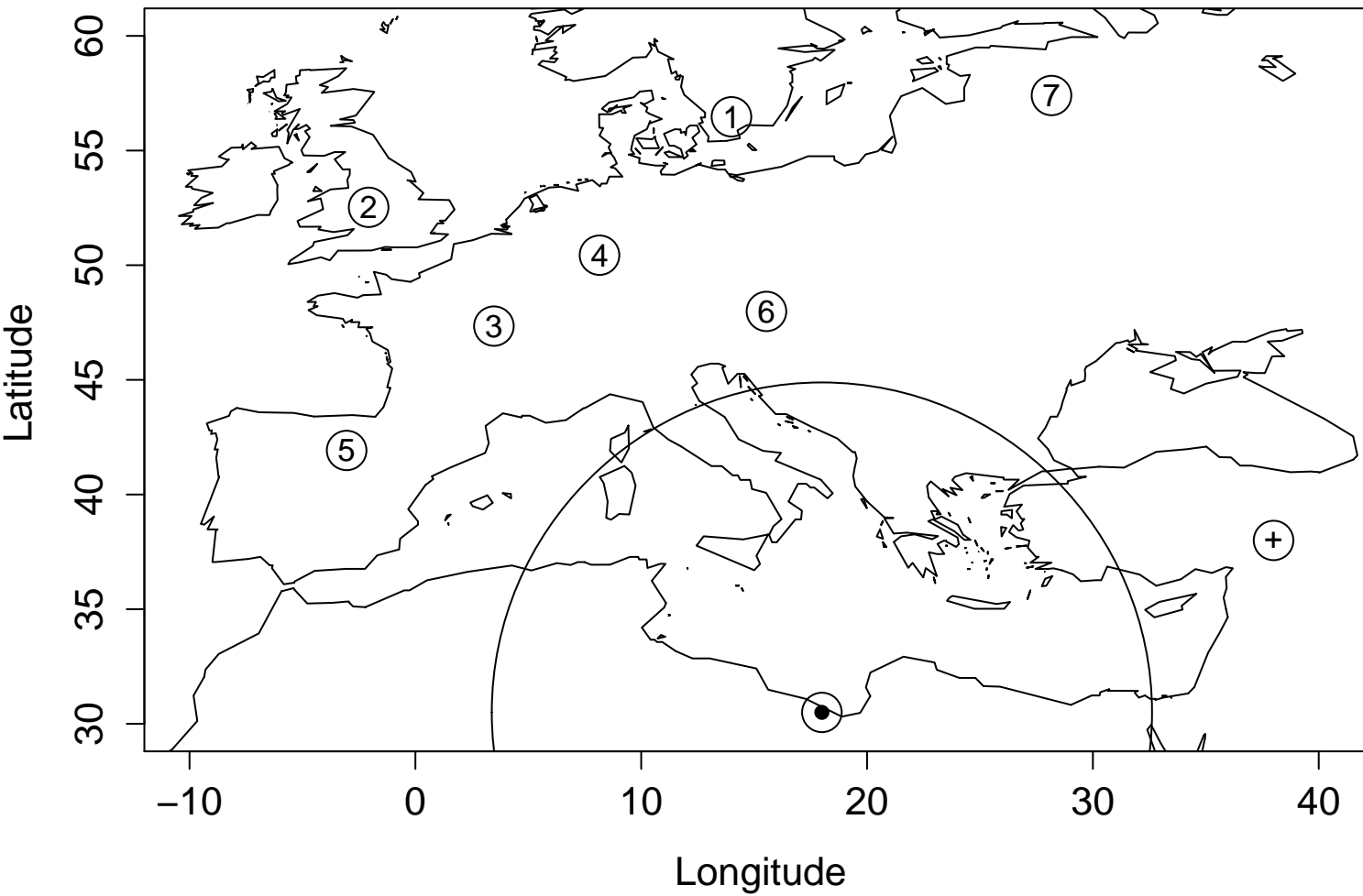

Supplement: Figure S2 — Sensitivity of the regression analysis to the geographic sampling scheme. The analysis was based on geographically explicit simulations using the computer program SPLATCHE. We assumed a date of onset of spatial expansion 10,000 years ago, carrying capacities in the interval (100, 5,000), migration rate m = 0.25, and growth rate r = 0.6. An Anatolian origin for the expansion was assumed, the origin was located at latitude 38°N and longitude 38°E, and was represented by a cross symbol in the figure. We generated 10 replicates of the simulation scenario, and, for each simulated data set, we inferred the most probable location for a putative origin by optimizing the R 2 statistic calculated in the regression of diversity on distance to the putative origin. The sampling scheme was identical to the one used to collect the actual data. The sample barycenter locations were 1: Southern Sweden, 2: British Isles, 3: France-Belgium, 4: Germany, 5: Iberia, 6: Central Europe, 7: Northeastern Europe (Table S2). The large circle surrounds the positions of the ten inferred origins, and the black dot represents their average position. See Text S1 for a more detailed discussion. (.04 MB PDF) [file pgen.1000075.s002.pdf]

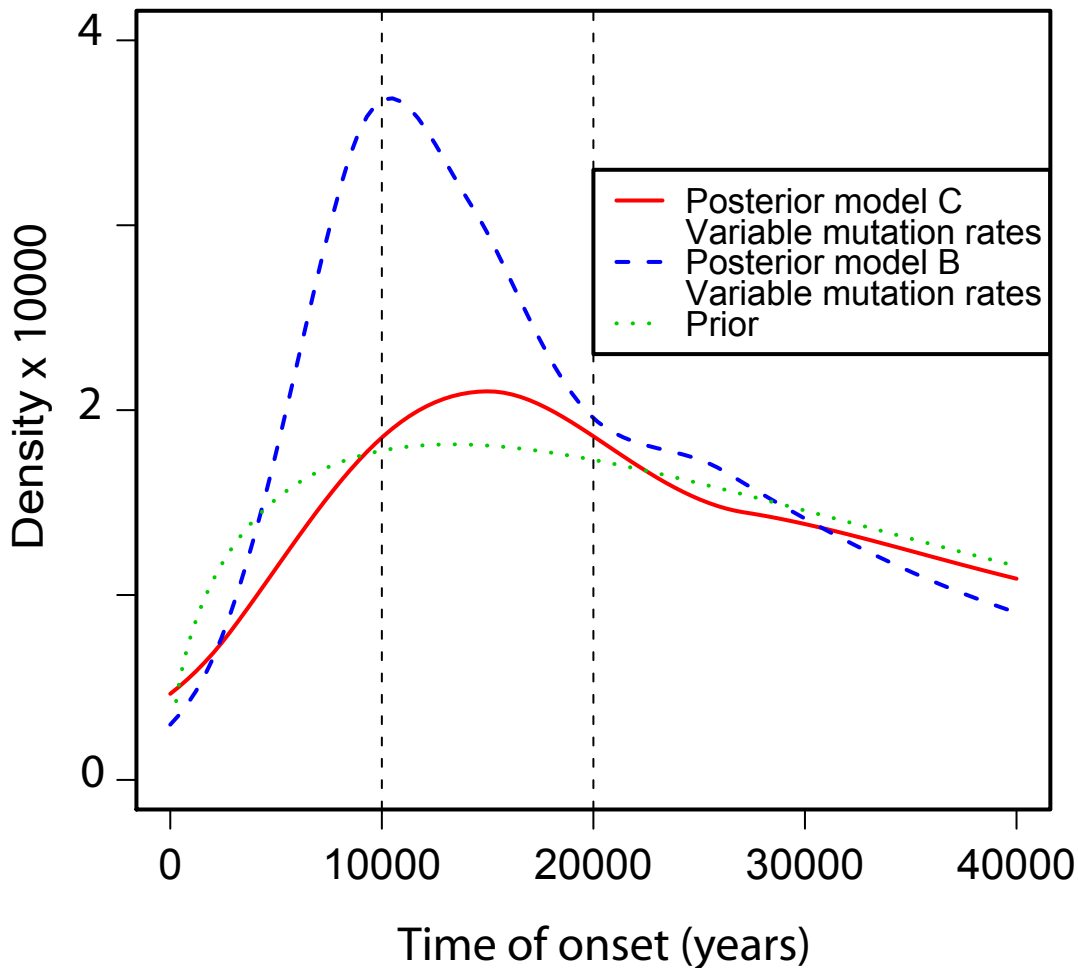

Supplement: Figure S3 — Posterior distribution for the time N 0 since the beginning of the expansion. The red solid line corresponds to Model C, for which the population size was initially constant, then grew exponentially from time t 0 to time t 1, and was constant again until the present. The dashed blue line corresponds to model B, for which the population size was initially constant, and then grew exponentially until the present. (.07 MB PDF) [file pgen.1000075.s003.pdf]

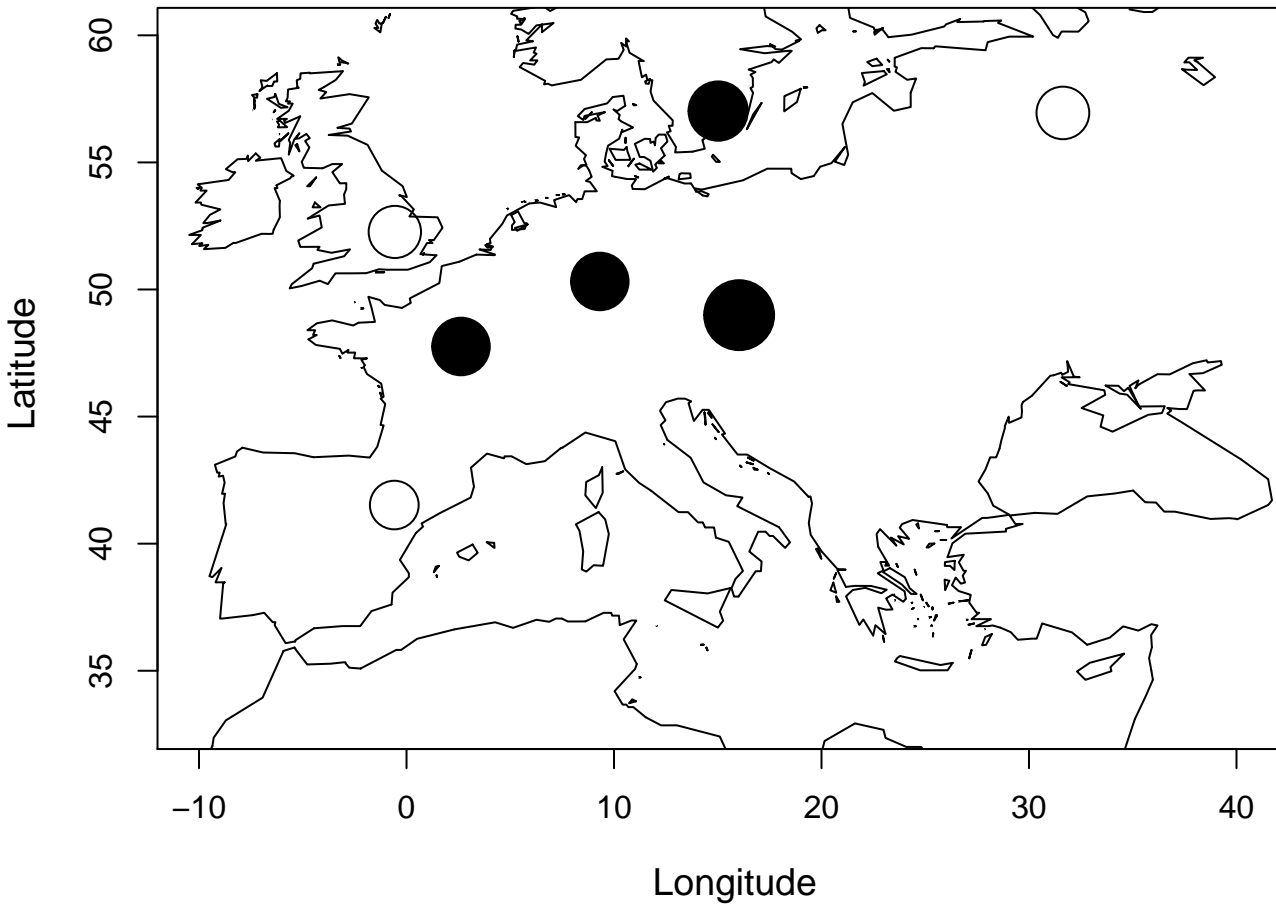

Supplement: Figure S4 — Mean number of distinct haplotypes in the seven samples used in the regression analysis. Higher values are in black circles, lower values are in white circles, and circle diameter is proportional to the mean number of distinct haplotypes. Exact values: Southern Sweden: 2.80, British Isles: 2.59, France/Belgium: 2.72, Germany: 2.72, Iberia: 2.41, Central Europe: 3.30, Eastern Europe: 2.61. See Table S2 for a description of the samples. (.02 MB PDF) [file pgen.1000075.s004.pdf]
