# Supplementary material for: Demographic History of European Populations of Arabidopsis thaliana
Source: PLoS Genet. 2008 May 16;4(5):e1000075. doi: 10.1371/journal.pgen.1000075 (PMC2364639; doi:10.1371/journal.pgen.1000075)
Supplement: Table S1 — List of 76 accessions used in the study. The geographic coordinates of Pu2-7 and Pu2-23 have been corrected to 49.42°N and 16.36°E (M. Nordborg, personal communication). See Nordborg et al. [21], Tables S1 and S2, for complete information about population samples and stock center accessions. (.02 MB PDF) [file pgen.1000075.s005.pdf]

1	Mr-0	39	Spr1-2
2	Bur-0	40	Spr1-6
3	Fei-0	41	Ull2-3
4	C24	42	Ull2-5
5	CICB-17	43	Omo2-1
6	CICB-5	44	Omo2-3
7	NFA-10	45	Var2-1
8	NFA-8	46	Var2-6
9	HR-10	47	Ler-1
10	HR-5	48	Ct-1
11	Sq-1	49	Uod-1
12	Sq-8	50	Uod-7
13	Edi-0	51	Bor-1
14	Ren-1	52	Bor-4
15	Ren-11	53	Lp2-2
16	Lz-0	54	Lp2-6
17	LL-0	55	Zdr-1
18	Pro-0	56	Zdr-6
19	Ts-1	57	Ws-0
20	Ts-5	58	Ws-2
21	Ag-0	59	CS22491
22	Se-0	60	Ms-0
23	Gy-0	61	Wa-1
24	Ra-0	62	Est-1
25	An-1	63	Pu2-23
26	Nok-3	64	Pu2-7
27	Mrk-0	65	Mt-0
28	Mz-0	66	Oy-0
29	Gu-0	67	Eden-1
30	Ei-2	68	Eden-2
31	Ei-0	69	Fab-2
32	Wt-5	70	Fab-4
33	Wei-0	71	Lov-1
34	Got-22	72	Lov-5
35	Got-7	73	Bil-5
36	Nd-1	74	Bil-7
37	Bay-0	75	Tamm-2
38	Br-0	76	Tamm-27
