# Supplementary material for: Demographic History of European Populations of Arabidopsis thaliana
Source: PLoS Genet. 2008 May 16;4(5):e1000075. doi: 10.1371/journal.pgen.1000075 (PMC2364639; doi:10.1371/journal.pgen.1000075)
Supplement: Table S2 — List of 7 samples used in the regression analysis of diversity on great circle distance. The samples were defined on the basis of geographic criteria. We corrected for the fact that the German sample contains twice the number of accessions present in France, Iberia, or eastern Europe by randomly sampling 6 accessions in this population, and we averaged heterozygosity over 100 replicates. The British Isles, Central Europe, and southern Sweden contain pre-defined populations consisting of more closely related individuals. (.01 MB PDF) [file pgen.1000075.s006.pdf]

Southern Sweden	Var2-1 Var2-6 Spr1-2 Spr1-6 Omo2-1 Omo2-3 Ull2-5 Ull2-3
British Isles	HR-5 HR-10 NFA-8 NFA-10 Sq-1 Sq-8 CIBC-5 CIBC-17 Edi-0
France/Belgium	Ag-0 Gy-0 Ra-0 Lz-0 Ren-1 Ren-11 An-1
Germany [6 among 10]	Bay-0 Mrk-0 Nd-1 Ga-0 Wt-5 Mz-0 Ei-2 Gu-0 Got-7 Got-22
Iberia	Se-0 Ts-1 Ts-5 LL-0 Pro-0 Fei-0
Central Europe	Uod-1 Uod-7 Zdr-1 Zdr-6 Bor-1 Bor-4 Lp2-2 Lp2-6 Pu2-7 Pu2-23 Br-0
Eastern Europe	Wa-1 Ws-0 Ws-2 Ms-0 Est-1 Ler-1
