# Supplementary material for: Demographic History of European Populations of Arabidopsis thaliana
Source: PLoS Genet. 2008 May 16;4(5):e1000075. doi: 10.1371/journal.pgen.1000075 (PMC2364639; doi:10.1371/journal.pgen.1000075)
Supplement: Table S3 — Prior distributions of parameter values under the various demographic models used during the ABC analysis. The parameter N 0 is the present population size, N 1 is the population size at the onset of expansion, r is the exponential growth rate (that is, the population size at time t before present is N(t) = N 0 e−rt ), t 0 is the time since the start of the expansion, and t 1 is the time since population size reached an equilibrium value. Time is measured backwards and in coalescent units of N 0 generations. LN denotes the log-normal distribution, and Г stands for the Gamma distribution. (.21 MB PDF) [file pgen.1000075.s007.pdf]

			Model parameters			
	μ ($\times 10^{-8}$)	N_0	t_0	r	t_0/t_1	N_2/N_1
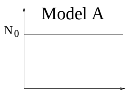	Exp(5)	LN($m = 5$, $\sigma = 0.5$)				
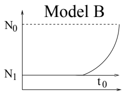	Exp(5)	LN($m = 5$, $\sigma = 0.5$)	$\Gamma(4 \times 10^4/3, 3/2)$	Exp(100)		
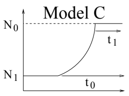	Exp(5)	LN($m = 5$, $\sigma = 0.5$)	$\Gamma(4 \times 10^4/3, 3/2)$	Exp(100)	Unif(0, 1)	
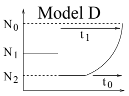	Exp(5)	LN($m = 5$, $\sigma = 0.5$)	$\Gamma(4 \times 10^4/3, 3/2)$	Exp(100)	Unif(0, 1)	Unif(0, 1)
