# Supplementary material for: Demographic History of European Populations of Arabidopsis thaliana
Source: PLoS Genet. 2008 May 16;4(5):e1000075. doi: 10.1371/journal.pgen.1000075 (PMC2364639; doi:10.1371/journal.pgen.1000075)
Supplement: Table S4 — Posterior distributions in the ABC analysis. Estimates of parameter values under four demographic models and their variants with variable mutation rates. For each parameter, the MAP estimate is followed by the 95% credibility interval. (.11 MB PDF) [file pgen.1000075.s008.pdf]

	Model parameters						
	μ ($\times 10^{-8}$)	N_0 ($\times 10^3$)	t_0 ($\times 10^3$)	N_1 ($\times 10^3$)	N_1/N_0	t_1 ($\times 10^3$)	$b = N_2/N_1$
Model A<br>Fixed mut. rate	1.6(0.3, 14.2)	104(39,1333)	-	-	-	-	-
Model B<br>Fixed mut. rate	1.2(0,10.2)	146(67,1815)	7(1,114)	0(0,823)	0(0,0.9)	-	-
Model C<br>Fixed mut. rate	1.6(0.6,10)	111(55,1172)	13(4,99)	0(0,409)	0(0,0.6)	4(0,56)	-
Model D<br>Fixed mut. rate	1.6(0.4,10.7)	169(63,146)	9(3,68)	105(24,922)	.9(.2,1)	20(4,1605)	1(0,1)
Model A<br>Variable mut. rate	2.4(0.6, 14.9)	265(66,1279)	–	–	–	–	–
Model B<br>Variable mut. rate	2(0.9,12.2)	179(65,1808)	10(4,108)	76(9,474)	0(0,0.6)	–	–
Model C<br>Variable mut. rate	2.2(1.1,11.2)	137(72,1228)	12(5,117)	0(0,447)	0(0,0.6)	5(0,80)	–
Model D<br>Variable mut. rate	2.3(0.6,13.4)	169(80,1552)	11(2,101)	116(39,968)	.9(.2,1)	22(5,2284)	1(0.1,1)
