# Supplementary material for: Demographic History of European Populations of Arabidopsis thaliana
Source: PLoS Genet. 2008 May 16;4(5):e1000075. doi: 10.1371/journal.pgen.1000075 (PMC2364639; doi:10.1371/journal.pgen.1000075)
Supplement: Table S5 — Bayes factors. The Bayes factors correspond to the ratio of the weight of evidence of each model to the weight of evidence of the variant of Model B with variable mutation rates. Two window sizes (or tolerance errors), δ 0.01 and δ 0.05, were used when computing the Bayes factors. These window sizes correspond to the 1% and 5% quantiles of the distance between observed summary statistics and the summary statistics obtained under the variant of Model B with variable mutation rates. (.02 MB PDF) [file pgen.1000075.s009.pdf]

	Bayes factor			
	Model A	Model B	Model C	Model D
Fixed mut. rate				
$\delta_{0.01}$	0	0.1	0.2	0.1
$\delta_{0.05}$	0	0.5	0.9	0.4
Variable mut. rate				
$\delta_{0.01}$	0	1	1.9	0.6
$\delta_{0.05}$	0	1	1.8	0.7
