# Supplementary material for: Demographic History of European Populations of Arabidopsis thaliana
Source: PLoS Genet. 2008 May 16;4(5):e1000075. doi: 10.1371/journal.pgen.1000075 (PMC2364639; doi:10.1371/journal.pgen.1000075)
Supplement: Text S1 — Supplementary text. (.08 MB PDF) [file pgen.1000075.s010.pdf]

Supplementary Text

Sensitivity analysis. The method used for locating the origin of the wave of expansion was based on the regression of diversity on geographic distance, following Ramachandran et al. (2005). Assuming a unique origin and recurrent founder effects, the model predicts an approximate linear decrease in diversity from the source population. We assessed the error generated by this method for the particular sampling scheme of the *A. thaliana* accessions by using spatially explicit simulations as performed by the software SPLATCHE. In simulated replicates, we sampled synthetic data at geographic locations that were identical to those present in the actual data set. These data were clustered into eight groups, seven of which corresponded to the samples used in the regression analysis for the real data set. The seven samples were defined on the basis of geographic and genetic proximity, with a balance between pooled individual accessions and actual population samples. The seven samples were labeled as (1): Southern Sweden, (2): British Isles, (3): France-Belgium, (4): Germany, (5): Iberia, (6): Central Europe, (7): Northeastern Europe. All remaining accessions were grouped into an eighth cluster, not used during the reanalysis.

In calibrating simulations from an expansion scenario, we used the demographic parameters estimated when fitting simulated folded frequency spectra to the *A. thaliana* data. For ease of simulation, multilocus genotypes were simulated according to the stepwise mutation model at 80 unlinked loci. Although these markers do not correspond to the data obtained for *A. thaliana* (DNA sequences), the choice of the marker type has a relatively minor impact on the conclusions of the regression study. We measured diversity in each sample as the variance in repeat numbers, averaged over the 80 loci. The mutation rate was fixed so that the levels of polymorphism were

on average comparable with those of *A. thaliana*. The sensitivity analysis assumed an Anatolian origin for the expansion, and the date of onset was 10,000 years ago. The origin was located at latitude 38 ° N and longitude 38 ° E, represented by a cross symbol in Figure S2. We generated 10 replicates of the simulation scenario, and for each simulated data set, we inferred the most probable location for a putative origin by optimizing the R^2 statistic calculated in the regression of diversity on distance to the putative origin. Geographic distances were computed as proportional to Euclidean distances in the map coordinates. In the same manner as in the analysis of real data, we did not use waypoints in computing geographic distances.

The ten best-fitting estimated origins were located in an area to the southwest of the true origin, showing that the particular geographic sampling used in the analysis might have been responsible for a significant estimation bias. The area containing the ten origins is roughly represented by a large circle in the south of the map in Figure S2. Here, the circled black dot represents the best-fitting origin obtained when pooling the ten data sets (the pooled data set may then be viewed as a single data set with 800 loci). The putative origin was located at latitude 31 ° N and longitude 18 ° E. The shift may be explained by the fact that the sample locations (5,3,4, and 6) were almost equidistant to this particular point, and displayed close levels of diversity. The samples (2, 1, and 7) formed a second equidistant ring, and an estimated origin to the west-southwest to the true origin provided significant discrimination between the two rings.

Four (of ten) estimated origins lay in an area compatible with the best-fitting location found for *A. thaliana*. This area was located close to the south of the Balkan region. Correcting for the observed bias in the analysis of the *A. thaliana* data would provide further evidence that the origin of spatial expansion, if it is unique, should be placed at the east of the Balkan region. The simulation study also indicates that the regression method perhaps has low power to detect a precise location of origin for the very peculiar design of the geographic sampling in *A. thaliana*.

Accession grouping and population designations. Given the mixed sampling of population samples and isolated ecotypes for *A. thaliana*, another conclusion from the simulation study is that the elevated levels of diversity in France (3), Germany (4) and central Europe (6) may be considered as expected results. The lower diversity in the British Isles (2), the southern Sweden sample (1), and Iberia (5) are also expected.

Removing the Finnish Tamm-2 and Tamm-27 accessions from the north-eastern European sample, the mean number of pairwise differences dropped from 0.0032 to 0.0029 in this sample. The Pearson coefficient R corresponding to the Balkan area decreased in absolute value from -0.50 to -0.45. In this case, it should be noticed that the decrease in correlation was better explained by the fact that removing the Finnish accessions changed the barycenter of the eastern group than by the mere change in diversity.

The most problematic sample was the sample from Germany (4), representing one of the largest geographic areas among the seven samples. The original data include 10 isolated accessions from Germany (Ei-2 Gu-0 Bay-0 Mrk-0 Got-7 Got-22 Ga-0 Mz-0 Wt-5 Nd-1). Compared to smaller samples (half the number of accessions in France, Spain, East Europe) or to population samples (Southern Sweden, Czech Republic, United-Kingdom), diversity in the German sample was inflated when the 11 accessions were included. To obtain comparably normalized results, it was necessary to correct for this bias and to drop 4 of the 10 German accessions. The map displayed in Figure 2 is computed with diversity in the German sample averaged over the 210 possible combinations of 6 German accessions. Accounting for the irregular sampling in *A. thaliana*, the estimated origin might then be considered to be actually biased westward.
